# Gemella morbillorum- and Capnocytophaga sp.-Related Mycotic Thoracic Aortic Aneurysm and Mediastinal Abscess: An Unusual Case Report, a Treatment Challenge, and a Review of Literature

**DOI:** 10.7759/cureus.17728

**Published:** 2021-09-05

**Authors:** Mina Said, Ekta Tirthani

**Affiliations:** 1 Internal Medicine, Rochester Regional Health, Rochester, USA

**Keywords:** gemella morbillorum, capnocytophaga, mycotic aneurysms, mediastinal abscess, antibiotic selection

## Abstract

A thoracic mycotic aortic aneurysm is an uncommon entity that can complicate mediastinal abscesses. *Gemella morbillorum* and *Capnocytophaga sp*. are oral bacteria that are very rarely encountered in this setting, especially when occurring together and with other organisms, posing a difficult treatment challenge per the available guidelines and sensitivities. We present in detail this interesting case of a multi-organism mediastinal abscess and thoracic mycotic aortic aneurysm after a previous esophagogastroduodenoscopic procedure in a 51-year-old female with known achalasia who presented with upper abdominal pain, including a successful surgical and antibiotic treatment regimen and a literature review of the involved topics.

## Introduction

A thoracic aortic mycotic aneurysm is an uncommon entity that can complicate mediastinal abscesses. *Gemella morbillorum* and *Capnocytophaga sp.* are common oral bacteria that are very rarely encountered in this setting especially when occurring together and with other organisms. This poses a difficult treatment challenge per the available guidelines and sensitivities. We present a case of a mediastinal abscess and mycotic thoracic aortic aneurysm after an esophagogastroduodenoscopic procedure caused by *Gemella morbillorum*, *Capnocytophaga sp.*, and *Streptococcus intermedius* that was successfully treated surgically and with an ertapenem plus gentamycin course followed by a step-down amoxicillin-clavulanic acid course. We also included a literature review of the involved topics.

## Case presentation

**Figure 1:**
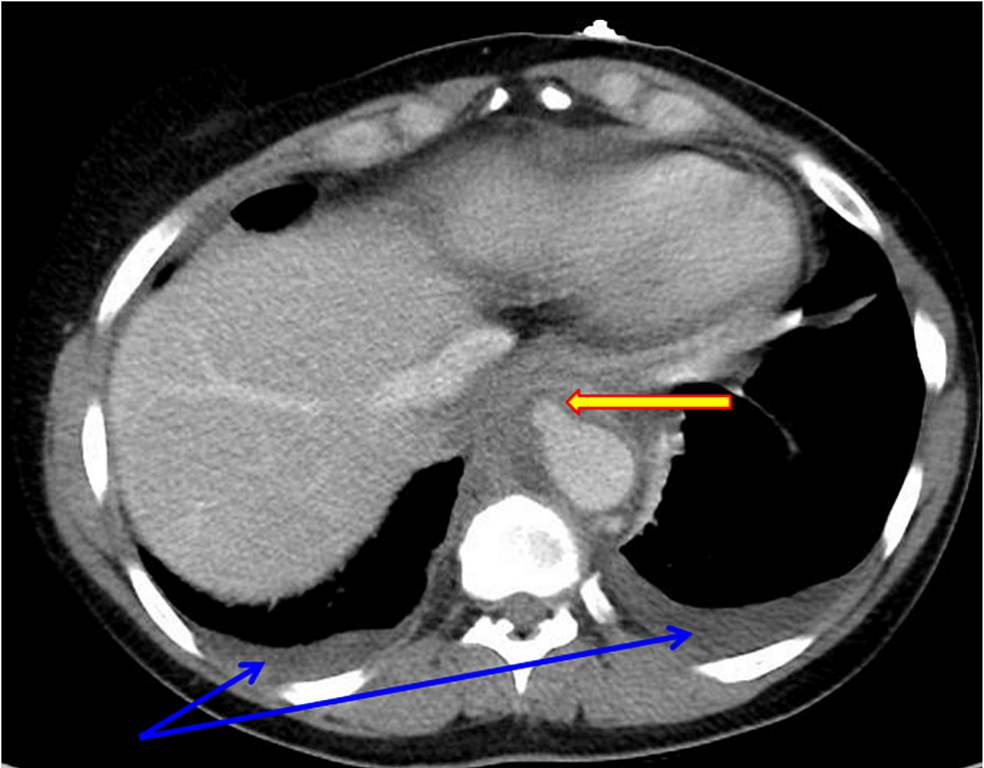
Abdominal CT scan with marked details CT abdomen showing a 1 cm saccular thoracic aortic aneurysm just below the diaphram with adjacent circumferential low density material (yellow arrow) and bilateral mild pleural effusions (blue arrows)

A 51-year-old Caucasian female with a known past medical history of achalasia and spinal stenosis had recently experienced heartburn and dysphagia to solids and liquids. Therefore, she underwent an oesophagogram study which revealed recurrent achalasia. That was followed by an esophagogastroduodenoscopy (EGD) that showed a dilated tortuous esophagus with some retained fluid. This prompted Botox injections above the gastro-oesophageal junction, a 20 mm balloon dilatation of the lower oesophageal sphincter, and a random biopsy showing mild chronic inflammation without malignant cells. She presented to the hospital 10 days later with a four-day history of progressive upper abdominal pain, mainly over the right upper quadrant, radiating to her back, sharp in quality, up to 8 out of 10 in intensity, without any specific alleviating or aggravating factors, but was associated with nausea, low appetite, intermittent fevers up to 39.1°C with diaphoresis and chills. Her presentation was also preceded by increased middle back pain for one week, that felt different and higher than her known lumbar spinal stenosis pain, and was severe enough that she was not able to lie on her back, for which she tried newly prescribed cyclobenzaprine, heat patches, ibuprofen, and even oral methylprednisone without much relief. She denied any change in body weight, chest pain, shortness of breath, cough, vomiting, hematemesis, melena, change in bowel habits, urinary symptoms, jaundice, numbness, weakness, or incontinence. Her past surgical history was notable for a laparoscopic Heller myotomy around 12 years before, multiple upper endoscopic oesophageal dilatations, and bilateral breast implants. She had no significant dental history. Her chronic medications included pantoprazole, gabapentin, and medroxyprogesterone. She used to drink alcohol only occasionally and did not smoke or use any recreational drugs. She lived in a house with her husband and three cats with no reported scratches or bites. She worked in the automotive industry and was independent in her daily activities. She was very active with her exercise routine and used to run marathons frequently. She had no recent travel or trauma. Her vital signs showed a blood pressure of 127/74, heart rate of 82 bpm, respiratory rate of 20/min, a fever of 38.6°C, and oxygen saturation of 95% on room air. On physical examination, she appeared uncomfortable, with tenderness over the epigastric and right upper abdominal quadrants without rebound tenderness, masses, Murphy’s sign, costovertebral angle, or spinal tenderness. Her initial workup showed a white blood cell (WBC) count of 28.9 x10^9/L mostly neutrophils and with 2% bands and toxic granulations present, normal hemoglobin of 13.2 g/dl, thrombocytosis of 531 x10^9/L, clear urine analysis. Her complete metabolic panel was normal apart from an alkaline phosphatase level of 127 U/L. Her lipase, lactic acid, beta-human chorionic gonadotropin (HCG), troponin level, and viral hepatitis panel were all unremarkable. She underwent blood cultures and severe acute respiratory syndrome coronavirus 2 (SARS-CoV-2) testing which were both negative. For imaging, she had a right upper quadrant ultrasound which showed a normal gallbladder, liver, and biliary tree. Chest X-ray suggested only mild right basal atelectasis. A CT of the abdomen and pelvis with IV contrast revealed a 1 cm saccular outpouching of the thoracic aorta just above the diaphragm with circumferential low-density material, concerning for a mycotic aneurysm, and small pleural effusions (Figure 1).

**Figure 2:**
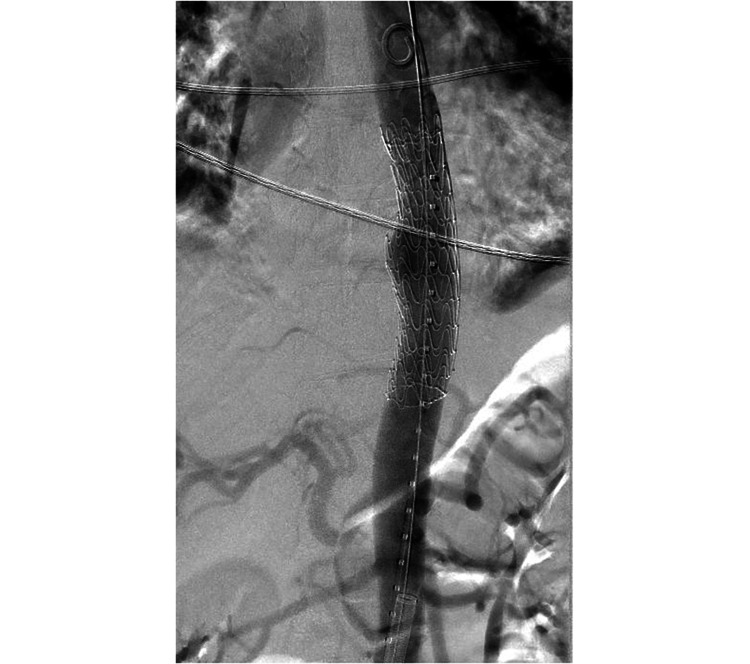
Temporary percutaneous endovascular repair of the mid descending thoracic aortic aneurysm with a stent graft

**Figure 3:**
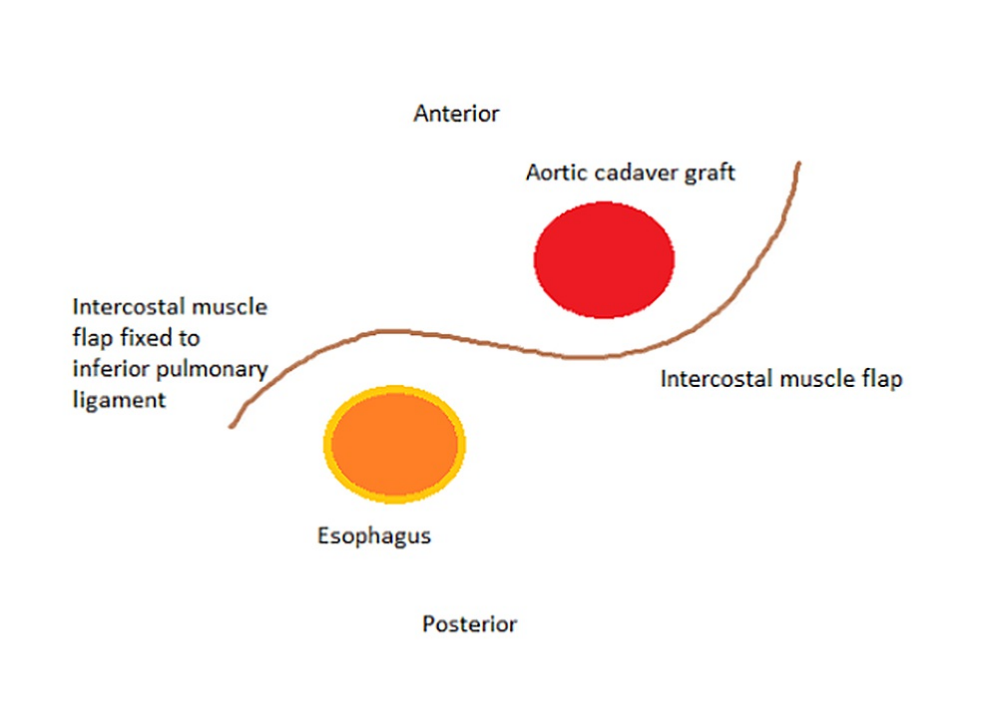
Graphic representation of the thoracic aortic aneurysm repair with removal of the endovascular graft and replacement with cadaveric graft, as well as a protecting intercostal muscle flap between the aorta and oesophagus

**Figure 4:**
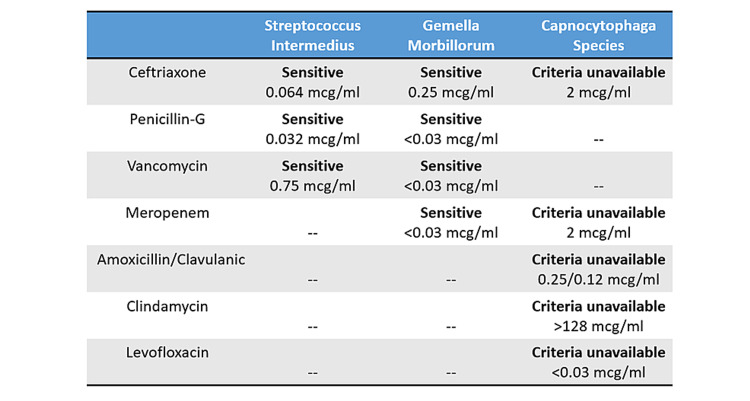
Summary of the sensitivities of the grown organisms on bacterial cultures

She was started empirically on vancomycin, piperacillin-tazobactam, and caspofungin for sepsis and possible oesophageal perforation. The infectious diseases team were consulted. She was taken urgently for a temporary percutaneous endovascular repair of the mid descending thoracic aorta with a stent graft (Figure 2). For the following three days, she continued to have moderate back pain and intermittent fevers up to 39.8°C with shaking chills, while her WBC came down to 15.4 x10^9/L and another repeat blood culture still showed no growth. Given the persistent fevers, lack of robust clinical improvement, and the need for better infection source control, the decision was made by the cardiothoracic surgery team for an open thoracic aortic repair. Therefore, on admission day five, she underwent a preoperative oesophagogram with no evidence of an oesophageal leak, and an upper endoscopy with a small site of an apparently healed oesophageal mucosal perforation on top of a pulsating bulge at 35 cm from the incisors. She then underwent a planned open thoracic aortic aneurysm repair by both cardiothoracic and vascular surgery with removal of the endovascular graft and replacement with cadaveric graft, as well as a protecting intercostal muscle flap between the aorta and oesophagus (Figure 3), with a notable intra-operative finding of posterior aortic wall defect, an adjacent mediastinal abscess with purulent material close to the intact oesophageal wall, and empyema which were debrided and sent for cultures, and two left chest tubes were kept in place, along with a nasogastric tube to prevent oesophageal dilatation. The periaortic fluid gram stain showed moderate (3+) WBC, rare gram-positive cocci, and gram-negative bacilli, while the pleural fluid showed many (4+) WBC and rare gram-negative bacilli. She remained on hydromorphone and acetaminophen as needed for her pain and low-grade fevers respectively, which both gradually subsided over the next few days. Her postoperative leukocytosis and chest tubes output also gradually improved. A repeat oesophagogram remained negative for an air leak so she was allowed to restart a regular diet. On day eight, the periaortic cultures started growing *Streptococcus intermedius*, and vancomycin was discontinued. By day 11, her periaortic cultures grew additional *Gemella morbillorum* from the anaerobic culture and *Capnocytophaga *species from liquid medium only, while the pleural fluid cultures remained negative. Chest tubes were removed, a peripherally inserted central catheter (PICC) line was placed, and sensitivities were run on the isolates to determine the best regimen for this challenging combination of organisms. In the meanwhile, under the infectious diseases team guidance, on day 14, her antimicrobial regimen was switched to meropenem, and then to ertapenem on her hospital discharge for easier once-daily dosing, along with gentamycin and fluconazole. Later after discharge, susceptibility results showed *Gemella morbillorum* minimum inhibitory concentrations (MICs) to be 0.25 mcg/ml to ceftriaxone, <0.03 mcg/ml to meropenem, <0.03 mg/ml to penicillin-G, and <0.03 mcg/ml to vancomycin, while *Capnocytophaga* MICs were 0.25/0.12 mcg/ml to amoxicillin-clavulanate, 2.0 mcg/ml to meropenem, 2.0 mcg/ml to ceftriaxone, >128 mcg/ml to clindamycin, <0.03 mcg/ml to levofloxacin, and finally *Streptococcus intermedius* was sensitive to all ceftriaxone with MIC 0.064 mcg/ml, penicillin-G with MIC 0.032 mcg/ml, and vancomycin with MIC 0.75 mcg/ml (Figure 4). She successfully completed two weeks of ertapenem and gentamycin with close monitoring of her complete blood counts, complete metabolic panel, inflammatory markers, and gentamycin levels. She was then continued on ertapenem and the same fluconazole throughout for three more weeks. She continued to be stable clinically and on weekly lab results. PICC line was removed and she was switched over to step-down oral amoxicillin-clavulanic acid for four more weeks by her infectious diseases specialist. At a one-month follow-up after antibiotics completion, she remained healthy and was back to her usual daily activities.

## Discussion

Mycotic aortic aneurysm is an infectious degeneration of the aortic wall which may result from direct local spread, inoculation, bacteremic seeding, or septic embolization, and poses significant morbidity and mortality risks. Mediastinal abscess is a localized purulent infection that may primarily complicate thoracic surgery or trauma [1]. The genus *Gemella*, first established in 1961 [2], has nine species among which *G. haemolysans* and *G. morbillorum* are the most important [3,4]. *Gemella morbillorum* is a part of the upper respiratory tract flora, as well as the gastrointestinal and genitourinary systems [5]. It was first described in 1917 [6], initially called *Streptococcus morbillorum* then *Peptostreptococcus morbillorum* [7], and finally transferred to the current genus in 1988 based on DNA homology and 16S RNA [8,9]. On the other hand, *Capnocytophaga* genus was first isolated from a case of meningitis following a dog bite in 1976 [10], then was given its present name in 1989 due to its high CO2 requirement [11]. It also consists of nine species, out of which *C. canimorsus* is the main aggressive human pathogen, while most others are oral human commensals [12,13].

Mycotic aortic aneurysms are uncommon and constitute <2% of the total aortic aneurysms, with thoracic aneurysms being just a small fraction of them [14,15]. Mediastinal infections are also unusual, complicating a range of 0.4-5% of cardiac surgeries. *Staphylococcus aureus* is the most common pathogen encountered in both conditions, though polymicrobial infections and less common agents may be seen [1,16,17]. *Gemella morbillorum* infections are in general very rare [18,19], with some risk factors including IV drug abuse, alcohol abuse, diabetes mellitus, chronic kidney disease, pre-existing valvular or congenital cardiovascular disease, chronic obstructive pulmonary disease, colon cancer, inflammatory bowel diseases, colonoscopies, and steroid use [20-22]. *Capnocytophaga sp*. on the other hand, are known to be associated with dog and cat bites or exposures [23], and mainly cause severe infections in the immunocompromised hosts like those with neutropenia, hematologic neoplasms, alcohol abuse, cirrhosis, and asplenia, or in immunocompetent hosts with anatomical abnormalities [4,16]. Poor oral hygiene and dental procedures are also deemed to be risk factors for both infections [23,24], though a report has found that they are isolated in higher levels from the oral microbiota of healthy controls than those with periodontal disease [25], except maybe for oral lichen planus patients in particular [26]. However, our patient is unique as she did not have any of these aforementioned pre-existing risk factors Instead, she had a history of a recent common upper endoscopic procedure. Moreover, this combination of uncommonly encountered pathogens in such uncommon conditions together makes it a valuable teaching addition to the available literature of each entity by itself.

Though very uncommon, *Gemella morbillorum* is known for predominantly being encountered with infective endocarditis [21,22,27,28], with between 90 to 100 cases reported in the literature [8,22,27,29]. It was also isolated in about 15 cases of pleural empyema since 1996 [3,7,20], mainly related to oesophageal stricture or dysphagia through aspiration [3,28]. It has also been seen with cases of spondylodiscitis [14,30], septic arthritis [31], meningitis [32], retropharyngeal abscess [33], and more rarely mediastinal abscesses [33], brain abscesses [20,34], and with related mycotic aneurysms sometimes [1,14]. There is one report about a case of fatal *Gemella morbillorum* descending mediastinitis from Ludwig’s angina precipitated by an infected tooth [35], and another that described the first case of *Gemella morbillorum* mediastinitis and osteomyelitis that followed an ultrasound-guided fine needle aspiration of a posterior mediastinal lymph node via transoesophageal endoscopic approach (a relatively similar setting to our patient) [36], as well as two similar cases following sternal traumatic introduction [37,38]. *Capnocytophaga sp*. has the ability to produce extracellular degrading enzymes to human tissues and evade phagocytosis [13,16,39], and is known for causing bite wound infections and severe sepsis [24,40]. It has also been implicated in cases of periodontitis [24], gingivitis [16], mucositis, ophthalmic lesions [16], cholecystitis [41], paravertebral abscesses [42], septic arthritis, peripartum infections [43], and endocarditis [44]. It was rarely involved in pleural empyema and mediastinal abscess [24,45], like our patient’s case. There has been a case of a traumatic oesophageal rupture and *Capnocytophaga *empyema from a motor vehicle accident in an immunocompetent patient [46], as well as another similar infection after a laparoscopic Nissen fundoplication with a likely oesophageal micro-perforation [47], in addition to more than four other spontaneous separately reported cases [45,48-51]. Another case from 2005 describes the first mycotic aortic aneurysm by *Capnocytophaga canimorsus* [11]. This concise review reveals there are only less than 10 cases for each of these organisms reported separately, with no reported cases found involving their co-existence together, nor involving a preceding upper endoscopic Botox injection, dilatation, or wall biopsy like what our case experienced.

Regarding identification, *Gemella *is a catalase-negative, facultatively anaerobic, gram-positive coccus, that grows best on high partial CO2 pressure [18,27,40]. It is one of the nutritionally variant streptococci (similar to *Granulicatella sp*. and *Abiotrophia defectiva*). It may initially be identified as *Streptococcus viridans* based on colony morphology [52], and it also tends to decolorize rapidly and can be falsely identified as gram-negative on gram stain due to their thin cell walls [53]. It has lately been reported more frequently due to advances in laboratory detection techniques [19]. Identification occurs through matrix-assisted laser desorption ionization-time of flight mass spectrometry (MALDI-TOF MS), polymerase chain reaction (PCR), and sequence analysis of the 16S RNA [3,27,40,45,54]. Many times, cultures are negative and they are only diagnosed by PCR of the infected tissue [27]. Capnocytophaga is an oxidase-positive, catalase-positive, indole-negative, gram-negative bacillus [12]. It may be seen under the microscope as intracytoplasmic fusiform rods in neutrophils allowing for a presumptive diagnosis [12,55]. It is best grown on blood agar in an anaerobic, 5-10% CO2 enriched medium [11,40], and usually takes a median of seven days to grow on cultures [56], which can delay the right diagnosis, and may be responsible for the 25-50% mortality rate in related severe infections [44,45]. In our case, it took almost six days for the *Gemella *and *Capnocytophaga *to just be isolated, taking into account that she was already on broad-spectrum antibiotics for days during the operative sampling. Also, many times, both are a part of a polymicrobial infection which we can likely assume [20], including other oral flora as *Peptostreptococci*, *Prevotella*, *Fusobacteria*, *Streptococcus viridans*, enterococci, and *Streptococcus intermedius* (just like our case with the latter) [12,28,56]. Therefore, specimens related to possible oesophageal perforation should be cultured in anaerobic and micro-aerophilic atmospheres and kept for several days to maximize the detection of such fastidious slowly-growing organisms which are crucial not to be missed [56].

*Gemella sp*. isolates in literature have generally shown sensitivity to penicillin, ampicillin, amoxicillin, cefuroxime, cefoxitin, cefotaxime, ceftriaxone, ceftazidime, imipenem-cilastatin, vancomycin, teicoplanin, erythromycin, clindamycin, linezolid, chloramphenicol, tetracycline, levofloxacin, and rifampicin [7,21,27,28,33]. However, it is mostly resistant to metronidazole and trimethoprim [7]. Therefore, penicillin and ampicillin were considered of choice for extra-abdominal *Gemella *infections [20], although in more recent years, resistance to penicillin and macrolides has been increasingly reported [5,28,52,57]. The Infectious Diseases Society of America (IDSA) treatment guidelines recommend treating penicillin-sensitive *Gemella *endocarditis with four weeks of either penicillin-G or ceftriaxone, or two weeks of penicillin-G or ceftriaxone plus gentamicin (a similar combination to enterococcal endocarditis) [19,23,29]. Penicillin-resistant *Gemella *on the other hand, are still managed with a combination of four weeks of penicillin-G and two weeks of gentamycin. Prosthetic valve infections are recommended to be treated with six weeks of penicillin-G or ceftriaxone and sometimes with gentamycin especially for penicillin-resistant strains [23]. Penicillin-allergic patients in all those cases can be treated with four to six weeks of vancomycin accordingly [20,23,31]. Combinations of ampicillin/chloramphenicol, penicillin/rifampicin, and erythromycin/rifampicin have been used successfully before in some reports [8,14]. *Capnocytophaga sp*. are usually sensitive to beta-lactamase inhibitor combinations, carbapenems, linezolid, clindamycin, erythromycin, tetracyclines, chloramphenicol, ciprofloxacin, moxifloxacin, rifampicin, and trimethoprim-sulfamethoxazole, but resistant to vancomycin, aztreonam, polymyxins, fusidic acid, aminoglycosides, fosfomycin, metronidazole, trimethoprim, and glycopeptides [11,12,16,40,45,56,58,59]. Moreover, *Capnocytophaga* has more frequently shown beta-lactamase production activity posing challenging resistance [16,56]. Unfortunately, there are no MIC cut-offs or guidelines on the choice and duration of antibiotics for *Capnocytophaga *infections [23,45], which definitely warrants careful susceptibility testing and interpretation on isolates [16]. Expert opinions recommend primarily using beta-lactamase inhibitor combinations, with alternatives that include imipenem-cilastatin, clindamycin, linezolid, and moxifloxacin [13,16]. The first challenge in appropriately managing our case was the need for a regimen that would cover all three bacteria (*Gemella*, *Capnocytophaga*, and *Streptococcus intermedius*) for an endovascular aortic infection. The second challenge was the need for an easy suitable regimen that would be feasible in an outpatient setting for the several weeks of planned treatment. She was initially managed broadly, then the decision was made for a targeted regimen of ertapenem and gentamicin, although the guidelines for *Gemella *endovascular infections recommended differently. That was based on the limited available data supporting the use of carbapenems for other *Gemella* or *Capnocytophaga *infections, the sensitivities of the isolates, the preference to have an anaerobic coverage, and ertapenem's dosing convenience. According to our literature review, ertapenem has very rarely been used for any of the encountered pathogens especially as a part of a *Gemella *endovascular infection treatment course, which makes the success of treatment of our case with this regimen even more noteworthy.

## Conclusions

We presented a case of a thoracic mycotic aortic aneurysm and mediastinal abscess following an upper endoscopic procedure, which is an uncommon complication. The cultured organisms, including *Gemella morbillorum*, *Capnocytophaga sp.*, and *Streptococcus intermedius* represent a rare occurrence in this type of infection, especially together, and also a treatment challenge to cover all three pathogens for this kind of endovascular infection with a feasible yet effective course. We reported the successful usage of ertapenem and gentamycin in our patient in addition to a review of literature of any similar cases and previous sensitivities.
